# 

**Published:** 1908-06-15

**Authors:** 


### CONTENTS

Event and Comment - - - - - - - - 271

Combination Fillings, - - - *By J. J. Travis, D.D.S.* 276

Some Camparative Qualities of Oxyphosphate and Silicate Cements, - - - - *By R. B. Tuller, D.D.S.* 294

Specific Causes of Oxyphosphate Cement Disintegration, *By J. O. Keller, D.D.S.* 300

Sulpho-Carbolic Acid—A Valuable Drug, *By Elgin Ma Whinney, D.D.S.* 308

Indents - - - - - - - - - - - - - 311

Announcements - - - - - - - - - 321

